# Obstacles in “Time to Spine”: Challenges for the Timely Delivery of Acute Surgical Care for Patients with Traumatic and Non-Traumatic Spinal Cord Injury

**DOI:** 10.3390/healthcare12222222

**Published:** 2024-11-07

**Authors:** Karlo M. Pedro, Mohammed Ali Alvi, Michael G. Fehlings

**Affiliations:** 1 Division of Neurosurgery & Spine Program, Department of Surgery, University of Toronto, Toronto, ON M5T 1P5, Canada; karlo.pedro@uhn.ca (K.M.P.); mohammed.alvi@uhn.ca (M.A.A.); 2 Institute of Medical Science, University of Toronto, Toronto, ON M5S 1A8, Canada; 3 Division of Neurosurgery, Krembil Neuroscience Centre, Toronto Western Hospital, University Health Network, Toronto, ON M5T 2S8, Canada

**Keywords:** spinal cord injury, early surgery, secondary injury cascade, knowledge translation

## Abstract

Over the past three decades, advancements in our understanding of the pathophysiology of spinal cord injury (SCI) have underscored the critical importance of early treatment for both traumatic and non-traumatic cases. Early surgical intervention significantly improves outcomes by limiting the extent of secondary damage. Despite numerous studies highlighting the superior outcomes associated with early decompression surgery for patients with SCIs, hospital reviews reveal that less than 60% of patients undergo surgical decompression within 24 h of injury. This occurs despite consensus among physicians regarding the benefits of early surgery. Therefore, it is important to highlight the multifactorial causes of this knowledge to action discordance. This review aims to elucidate the administrative, logistical, and technical challenges that hinder timely access to surgery for SCIs.

## 1. Spinal Cord Injury: Recognizing the “Burden”

Spinal cord injury (SCI) encompasses physical, social, and economic dimensions and profoundly impacts patients and their families [1]. Despite significant progress in elucidating pathophysiological changes after SCI, effective neuro-regenerative therapies that can help SCI patients regain their neurologic and functional recovery remain elusive. According to global burden of illness research, there were 0.93 million cases of SCI in 2016, with an incidence rate of 13 per 100,000 people based on age [2]. Long-term disability and dependence on caregivers are major consequences of SCI. The combination of sensory and motor deficiencies, along with neurogenic sphincter dysfunction, severely impair a person’s quality of life and their capacity for autonomous and productive functioning, which is often compounded by depressive symptoms (in 20–30% of individuals with SCI) [1,3]. These statistics demonstrate the substantial financial, functional, and physical costs associated with chronic SCI.

## 2. Importance of “Early Surgery” in Acute SCI

Our understanding of SCI pathophysiology highlights primary and secondary injuries, triggering a chain reaction of endothelial, axonal, and neuronal dysfunctions [4]. The term “primary mechanism” describes the initial compression of the cord following a fracture, which sets off a series of pathobiological alterations that lead to secondary damage. Conversely, secondary mechanisms are defined as reversible alterations such as hemorrhage, edema, vasospasm, ischemia, excitotoxicity, and apoptosis that may result in additional spinal cord damage. Continuous compression against the inflexible spinal canal further damages neural tissues [5]. Both preclinical animal research and subsequent human studies demonstrate that early decompression to release the spinal cord improves long-term neurological and functional outcomes [6,7]. Early surgery reduces complication risks, shortens hospital stays, and lowers hospitalization costs [8]. Santos et al. modeled the indirect benefits of surgery within 24 h in a Canadian SCI facility, demonstrating improvements in quality of life, life expectancy, neurological recovery, and complication rates in tetraplegic patients [9]. One study found that among patients admitted for a complete cervical SCI, early surgery is linked to improved neurological status and an improvement in AIS grade [10]. Early surgery, particularly for cervical SCI, is considered best practice [11].

## 3. Knowledge to Translation Discordance

There is now sufficient evidence demonstrating the beneficial role of early surgery for acute SCI. In a recent systematic review and meta-analysis, Fehlings et al. found that, based on moderate evidence, patients undergoing early surgery were twice as likely to recover two grades or more on the ASIA Impairment Scale compared to those undergoing late surgery [12]. In another systematic review and meta-analysis by Perez et al., the authors analyzed six studies consisting of 1188 patients, of which 592 patients underwent early decompression. The authors found that patients in the early group were more likely to experience neurological improvement (an ASIA Impairment Scale improvement of two or more) at follow-up compared to those undergoing late surgery [13]. In another study by Shi et al., the authors queried records of two large academic medical centers in China for patients with traumatic cervical SCI. The authors found that incomplete SCI patients undergoing early surgery were significantly less likely to develop neuropathic pain [14]. Despite the evidence supporting early surgery, in the practical world, a number of circumstances may lead to delays in getting the patient to the operating room for surgical decompression. Less than 50% of SCI patients in North America receive early surgery following traumatic SCI [15,16,17,18]. Survey data indicate that patients admitted to academic teaching hospitals have a greater chance of receiving surgery within a day compared to non-academic centers, highlighting discrepancies in knowledge translation [19]. A 2017 survey revealed that most surgeons recognize the benefits of early surgery, yet practical challenges persist [18]. In another study from South-East Norway, Aarhus et al. queried a population-based database to analyze 243 patients with cervical SCI. The authors found that 47% of patients were able to undergo surgery within 24 h [20].

## 4. Inter-Hospital Transfer Delays

A common cause of delay is the initial admission of patients to local or community hospitals before they are transferred to specialized SCI or level 1 trauma centers. A higher-level trauma center’s resources are frequently needed for surgical intervention and the care that follows for traumatic SCI. Treatment at these centers is associated with lower complication rates and mortality, supported by features such as intensive care units, multidisciplinary teams, and advanced diagnostic and therapeutic capabilities [21,22]. A study by Thompson et al. conducted at a single level 1 trauma center in Quebec, Canada, found that 90.3% of patients were first transferred to a community hospital, resulting in longer delays to surgery [23]. Similarly, Kelly Hedrick et al. analyzed the North-American Clinical Trials Network (NACTN) data and found that 40.7% of patients were transferred to an NACTN facility (all level 1 trauma centers with specialized SCI care) from another hospital. Patients who were admitted directly to a NACTN facility had a median time to surgery of 22 h, whereas patients who arrived via inter-hospital transfer had surgery at a median of 36 h after the injury [24].

In a third study by Wilde et al., performed at a tertiary care center in Utah, USA, the authors analyzed patients undergoing rehabilitation care for SCI at their center after undergoing operative or nonoperative acute management. The authors found that 26.2% of patients were transferred from a community hospital. These patients had a duration of 2.7 days (64 h) between the time of injury and surgery compared to patients who underwent surgery at the tertiary care center (average duration of 1.7 days or 38 h) or another community hospital (average of 1.3 days, or 32 h) [25].

Furlan et al. analyzed 63 patients from a single institution in Toronto, of which 23 patients (36.5%) received early surgery while 40 patients (63.5%) underwent late decompression. The authors found that patients in the late surgery group had to wait significantly longer times in another general hospital (1982.1 min) compared to those in the early surgery group (577.6 min) [17].

In a study by Glenie et al., the authors conducted a survey involving Canadian surgeons in which they presented cases of acute traumatic SCI and asked about the ideal time from injury to SCI, as well as potential barriers that may exist in attaining such ideal surgical timing. Patient transportation was cited by almost one-third of respondents as a significant cause of the longer management times [18].

## 5. Administrative Delays

There may be some administrative reasons for delays in surgery as well. The study by Thompson et al. found that among patients who presented to a tertiary care center between Monday and Thursday, 50% were able to undergo the surgery, compared to 75% among those who presented to the center between Friday and Sunday [19]. Accessibility issues to specialist imaging, particularly magnetic resonance imaging (MRI), as well as the large volume of elective cases being handled concurrently during the week, means that the handling of trauma patients who arrive during the week potentially relies on the availability of operating rooms and spine surgeons [23]. The same study found that the specialty of the first MD who assessed the patient in the ER also impacted surgery timing. Patients assessed by trauma surgeons were more likely to receive early surgery than those assessed by spine surgeons, possibly due to trauma team prioritization. Similarly, Furlan et al. reported shorter wait times for spine surgeon evaluation (73.5 vs. 274.4 min) and surgical decision-making (241.7 vs. 832.3 min) in the early surgery group [17].

## 6. Pre-Hospital Care

Pre-hospital care guidelines are being increasingly recognized as needing end users’ input and comments to ensure their applicability. PHC interventions, as per current protocols, involve prompt triage for patients suspected of having SCI, extrication, initial resuscitation (protection of the airway and cervical spine, breathing, and circulation), and the immobilization of patients suspected of having an SCI at the scene, followed by prompt transport to a trauma center. However, according to a scoping review by Arejan et al., there is no consensus regarding spinal immobilization in patients who may have an SCI. In addition to being debatable when it comes to blunt spine injuries, recent research has questioned immobilization in cases of penetrating spinal injuries. Pre-hospital care clinicians can greatly enhance patient outcomes and prevent needless spinal immobilization by properly and promptly identifying possible SCIs [26].

Research on emergency medical service (EMS) personnel’s decision-making and the application of clinical decision criteria in pre-hospital and emergency situations is lacking. Understanding how protocols are interpreted and used in the field is necessary to enhance patient care as recommendations for spinal treatment continue to change. McDonald et al. examined EMS personnel’s opinions about present practices in light of a developing standard for spine care using a survey [27]. The survey’s findings indicated that paramedics’ opinions on pre-hospital SMR are multifaceted and varied. While survey respondents claim to adhere to pertinent protocols most of the time, in-depth answers show a variety of methods in which healthcare professionals strike a compromise between trying to maximize treatment and adhering to protocols. Most remarkably, replies showed widespread doubt about the benefits of spinal motion restriction. Expanding upon these findings, free-text replies offered particular instances of how paramedics maneuver around the practice setting and ease the stress that develops when prescribed care does not correspond with the clinical scenario. For instance, EMS personnel reported that spinal motion restriction can occasionally result in motion despite best efforts. Understanding the negative impacts of motion will enable the provision of recommendations for improvements and workarounds. Many respondents characterized SMR as ineffective, noting that practically all patients are aggravated by treatment devices, particularly those who are disturbed or nervous. The survey also noted additional techniques to decrease movement, which also mentioned the negative effects of pre-hospital spinal motion restriction. These included purposefully undersizing cervical collars, permitting different placements, and forgoing treatment when protocol demanded it. The ability to identify low-risk patients as candidates for further assessment without the use of restraining devices, the use of a soft cervical collar (as opposed to a rigid one), sedation options, allowing patients to self-extricate from vehicles, and the removal of the long backboard for transport were among the suggestions for improvements.

Taken together, these points indicate a need to optimize pre-hospital care for SCI patients that could streamline triage and funnel surgical candidates for SCI. The management of SCIs requires a coordinated and time-sensitive approach, similar to established stroke protocols. Improving pre-hospital care and patient flow for SCI patients is crucial for optimizing outcomes and reducing secondary complications [28]. Improper handling can exacerbate neurological deficits [29]. Implementing standardized assessment tools and treatment algorithms for pre-hospital personnel can enhance the early identification and management of potential SCI cases [30]. Establishing clear communication channels between emergency medical services and receiving hospitals allows for better preparation and resource allocation [31]. Creating designated SCI centers with specialized expertise and equipment, analogous to stroke centers, can improve patient outcomes by centralizing care. Implementing the “time is spine” approach, similar to the “time is brain” concept in stroke care, emphasizes the importance of rapid transport and early intervention [26]. Developing regionalized systems of care with pre-determined transfer protocols can ensure patients receive appropriate treatment in a timely manner. The continuous education and training of pre-hospital providers, emergency department staff, and other healthcare professionals involved in SCI care is crucial for maintaining high-quality, evidence-based practices. By adopting these strategies, healthcare systems can significantly improve their pre-hospital management and overall care pathway for SCI patients.

## 7. Patient Factors Necessitating Surgical Delays

In addition to logistical and administrative challenges, patient-specific factors can also necessitate delays in surgical intervention for SCIs. For instance, comorbid cardiac conditions may require stabilization before proceeding with surgery. Acute cardiac issues such as myocardial infarction, arrhythmia, or heart failure can significantly increase perioperative risk, necessitating preoperative optimization to avoid exacerbating these conditions. Polytrauma patients with severe concomitant injuries in other body systems may also preclude early surgical intervention to prioritize treatment of more life-threatening injuries. Furthermore, patients on anticoagulant therapy, particularly those taking novel anticoagulant factors (NOACs) or direct oral anticoagulants (DOACs), require careful management; specifically, close coordination with the hematologist and the hospital thrombosis team. These medications, essential for preventing thromboembolic events, necessitate a washout period to minimize the risk of perioperative bleeding [32,33]. Consequently, the need for cardiac and hematological optimization can contribute to delays in surgical decompression, emphasizing the importance of individualized SCI patient care, a team effort, and close coordination with critical care specialists.

## 8. Ethical Considerations in Frail and Elderly Patients

Complex ethical considerations may arise when treating very old and frail patients with severe SCI. The prognosis for elderly patients with significant comorbidities and severe neurological deficits is often poor, leading to difficult decisions regarding the appropriateness of aggressive surgical interventions [34]. In such cases, the potential benefit of surgery must be weighed against the risk of operative and postoperative complications, including prolonged hospitalization, increased morbidity, and the possibility of diminished quality of life. For some patients, particularly those with limited life expectancy and significant frailty, the focus may shift towards compassionate measures and palliative care. These considerations require a multidisciplinary approach involving discussions with patients, families, and healthcare teams to ensure shared decision-making aligned with the patient’s values and best interests [35].

## 9. Challenges to Early Surgery on a Global Scale

In the past decade, disparities in healthcare resources have been identified as a key issue for providing quality surgical care worldwide, including early decompression for SCIs. During the 68th World Health Assembly in 2015, the member states of the World Health Organization supported a unanimous call to “strengthen emergency and essential surgical care and anesthesia” in view of the Lancet Commission on Global Surgery report. This study showed that the surgical disease burden, including 5 million neurosurgical procedures per year, was greater than the problem of communicable diseases and could be addressed by collective efforts to improve surgical healthcare in low- and middle-income countries (LMICs) [36]. In a recent worldwide survey of spine specialists, it was estimated that in low-income countries (LICs), where approximately 650 million people live, only one out of six traumatic SCI patients are operated on within 24 h. In LMICs, where approximately 3.3 billion people live, one out of two surgeries for SCI are delayed for more than 24 h [37].

Irrespective of a country’s economic situation, transfer time is cited as the most common reason for surgical delay in the care of SCI patients [38]. Systemic issues, such as long transportation distances from remote areas, add another layer of complexity to the timely treatment of SCI in LMICs. Patients from these regions often face significant delays in reaching specialized medical centers equipped to handle SCI cases. Poor infrastructure, inadequate transportation systems, and geographical barriers exacerbate these delays [39]. Furthermore, the concept of “time is spine” is challenged by the high costs of surgery and the limited availability of surgical instruments, posing significant barriers to effective treatment. In the survey by Marchesini et al., around one-quarter of the world’s population is estimated to live in areas where the cost of an operation is completely covered by the patient or their families [37] without government subsidy. Additionally, the availability of surgical equipment and advanced spinal imaging is often limited, leading to failures in attaining the recommended early time cutoff for surgical intervention in SCI. Recent clinical trials in neurotrauma further highlight a unique and entirely different set of requirements in LMICs compared to high-income countries. Examples include the unavailability of intravenous fluids in far-flung areas or the lack of proper rescue and transport systems, which might not be the case in high-income regions [40]. Furthermore, although more than half of the total caseload of neurotrauma (traumatic brain and spinal cord injury combined) occurs in LMICs, many of these countries do not have a single neurosurgeon or have neurosurgeons practicing mainly in urban centers, further decreasing the prospect of early surgical care in remote areas [41]. This gamut of factors in low-resource regions, combined with higher incidences of neurotrauma, tremendously impacts the global community in terms of functional disability and death from these potentially survivable and salvageable surgical conditions. Addressing these issues requires targeted investments and international support to improve infrastructure, enhance transportation networks, and develop decentralized healthcare facilities to reduce travel times, improve surgical capacity, and access essential surgical equipment. Recognizing these systemic barriers is the crucial first step for improving SCI outcomes globally.

## 10. Conclusions

Timely surgical intervention for SCIs is crucial for minimizing secondary damage and improving long-term outcomes. Despite the clear benefits of early surgery, various administrative, logistical, and patient-specific factors contribute to delays. These include inter-hospital transfer issues, administrative scheduling conflicts, and the need for patient stabilization due to comorbid conditions like cardiac issues or anticoagulant therapy. Additionally, ethical considerations play a significant role in decision-making for frail, elderly patients with severe SCIs. On a global scale, particularly in LMICs, systemic challenges further complicate the timely delivery of surgical care for SCIs. There is certainly a need to investigate additional challenges that may prevent patients with SCIs from gaining access to early surgical care and how they may be minimized. Addressing these multifaceted barriers across the spectrum of local communities to the global scale requires a comprehensive trauma-team approach supported by national policies and international collaborations to ensure that more patients can benefit from early surgical decompression and achieve better clinical outcomes.

## Data Availability

No new data were created or analyzed in this study.
